# Biodegradation of Low-Density Polyethylene (LDPE) by Mixed Culture of *Lysinibacillus xylanilyticus* and *Aspergillus niger* in Soil

**DOI:** 10.1371/journal.pone.0071720

**Published:** 2013-09-23

**Authors:** Atefeh Esmaeili, Ahmad Ali Pourbabaee, Hossein Ali Alikhani, Farzin Shabani, Ensieh Esmaeili

**Affiliations:** 1 Soil Science Department, Faculty of Agricultural Engineering and Technology, University College of Agriculture and Natural Resources, University of Tehran, Karaj, Iran; 2 Ecosystem Management, School of Environmental and Rural Science, University of New England, Armidale, Australia; 3 Department of the Environment, Tehran, Iran; University of Kansas, United States of America

## Abstract

In this study, two strains of *Aspergillus* sp. and *Lysinibacillus* sp. with remarkable abilities to degrade low-density polyethylene (LDPE) were isolated from landfill soils in Tehran using enrichment culture and screening procedures. The biodegradation process was performed for 126 days in soil using UV- and non-UV-irradiated pure LDPE films without pro-oxidant additives in the presence and absence of mixed cultures of selected microorganisms. The process was monitored by measuring the microbial population, the biomass carbon, pH and respiration in the soil, and the mechanical properties of the films. The carbon dioxide measurements in the soil showed that the biodegradation in the un-inoculated treatments were slow and were about 7.6% and 8.6% of the mineralisation measured for the non-UV-irradiated and UV-irradiated LDPE, respectively, after 126 days. In contrast, in the presence of the selected microorganisms, biodegradation was much more efficient and the percentages of biodegradation were 29.5% and 15.8% for the UV-irradiated and non-UV-irradiated films, respectively. The percentage decrease in the carbonyl index was higher for the UV-irradiated LDPE when the biodegradation was performed in soil inoculated with the selected microorganisms. The percentage elongation of the films decreased during the biodegradation process. The Fourier transform infra-red (FT-IR), x-ray diffraction (XRD) and scanning electron microscopy (SEM) were used to determine structural, morphological and surface changes on polyethylene. These analyses showed that the selected microorganisms could modify and colonise both types of polyethylene. This study also confirmed the ability of these isolates to utilise virgin polyethylene without pro-oxidant additives and oxidation pretreatment, as the carbon source.

## Introduction

Synthetic plastics, such as polyethylene, are used extensively in packaging and other industrial and agricultural applications. These plastics are characteristically inert and are resistant to microbial attack, leading to their accumulation in the environment. Recently, the biodegradation of plastic waste and the use of microorganisms to degrade the polymers have gained notable importance because of the inefficiency of the chemical and physical disposal methods used for these pollutants, and the environmental problems they cause. Microorganisms play a significant role in the biological decomposition of material [1]. However, the high molecular weight, 3**-**dimensional structure, hydrophobic nature and lack of functional groups in the LDPE interfere with microbial attack. The generation of biodegradable PE (polyethylene) requires modifying the properties that are responsible for the PE resistance to degradation. The UV irradiation (photo**-**oxidation) and, thermal and chemical oxidation of PE prior to its exposure to a biotic environment enhances biodegradation [2]. These pretreatments increase surface hydrophilicity of the polymer by the formation of additional groups such as carbonyl groups that can be utilised by microorganisms [3], [4], [5]. Various methods are available to estimate the biodegradability of plastics. It is desirable to estimate the biodegradability of plastic wastes under natural condition such as soil [6]. A standard test to determine the biodegradation of plastic materials when exposed to soil was developed by the ASTM [7]. The microbial degradation process of polymers is initiated by the secretion of enzymes which cause a chain cleavage of the polymer into monomers. Metabolism of the split portions leads to progressive enzymatic dissimilation of the macromolecules from the chain**-**ends; eventually, the chain fragments become short enough to be consumed by microorganisms [8]. In most studies, fungi have been investigated for the biodegradation of LDPE because these organisms produce degrading enzymes [1] and, extracellular polymers, such as polysaccharides, which can help to colonise the polymer surface [9], and the distribution and penetration ability of the fungal hyphae is an advantage. Some studies have investigated the PE biodegradation process using fungal isolates, such as *Phanerochaete chrysosporium* [6], *Aspergillus niger* [9], [10], and other strains of the *Aspergillus* genus including *A. terreus*, *A. fumigatus* [11] and *A. flavus* [12]. There are reports in the literature confirming the ability of bacteria to degrade PE. Sivan et al. [13] isolated a biofilm**-**producing strain of *Rhodococcus ruber* (C208) that degraded PE at a rate of 0.86% per week. Hadad et al. [14] isolated a thermophilic bacterial strain (707), identified as *Brevibacillus borstelensis*, which utilised standard and photo-oxidised PE. The ability of *Bacillus* species to utilise PE, with and without pro**-**oxidant additives, was also evaluated [15]. In this study, selected microorganisms were isolated from a typical aged landfill and were identified as *Aspergillus niger* (designated F1) and *Lysinibacillus xylanilyticus* XDB9 (T) strain S7**–**10F. The ability of these isolates to degrade LDPE films in soil was investigated.

## Materials and Methods

### 1. Materials

An Iranian petrochemical company provided the low-density polyethylene granules (LF0200, with a density of 0.920 gr.cm^−3^) and the ethylene oligomer (C_20_ **–**C_40_). The LDPE films (20 µm thick) were made from the LDPE granules using a blowing film extruder.

### 2. Enrichment culture and isolation of microorganisms

The enrichment procedure was performed to isolate microorganisms that utilise PE as the sole source of carbon. Different soil samples (11 in total) were collected randomly from landfills in which PE wastes had been buried for different periods. In the remainder of this paper, no specific permissions were required for soil sampling or the described field studies done by the first author of this study. Also, it should be confirmed that the study area was not privately-owned and the field studies did not involve endangered or protected species.

The following 3 methods for the enrichment culture were performed using LDPE films and powder:

1. Soil samples, 10 g each, were placed in test tubes containing 4 ml of synthetic mineral medium containing (grams per litre): NH_4_NO_3_, 1.0; MgSO_4_.7H_2_O, 0.2; K_2_HPO_4_, 1.0; CaCl_2_.2H_2_O, 0.1, KCl, 0.15 and approximately 300 mg of polyethylene film. The test tubes were incubated for 20 weeks at 30°C [2].
2. Each soil sample (10 g) was placed in 250 ml Erlenmeyer flasks containing 50 ml of synthetic mineral medium, and 1 g of PE powder (LF0200) was added to each flask as the sole source of carbon. The cultures were incubated on a rotary shaker (120 rpm) at 30°C for 12 weeks.
3. This method is the same as method 2 except the flasks were incubated without shaking at 30°C for 12 weeks.

After termination of the enrichment procedure, the initial isolation of microorganisms was performed in solid media (synthetic mineral medium**-**agar) containing linear paraffin as the sole source of carbon, and the microorganisms were selected through growth comparisons. Next, the screening of the selected microorganisms was performed by comparing their growth ability in solid media containing 2% liquid ethylene oligomer as the sole source of carbon. The microorganisms that were selected were cultured in liquid mineral medium (synthetic mineral medium) containing different concentrations of liquid ethylene oligomer (3, 4 and 5%). The microorganisms with the ability to grow in the presence of 5% ethylene oligomer were transferred to synthetic mineral medium containing 0.1% PE powder as the sole source of carbon for the final screening step.

### 3. Identification of isolated microorganisms

The taxonomic identification of the bacterial isolate, including biochemical characterisation and PCR amplification of the 16S rDNA, was performed at the Iranian Biological Resource Center (IBRC). The partial nucleated sequence of the 16S rDNA from isolate S7**–**10F was determined by the Macrogen Co. in South Korea (using ABI system 3730 XL) and was deposited in the NCBI database under Genbank Accession No: JF838304.

The identification of the fungal isolate was performed by recognising the diagnostic morphological features of genera using macroscopic and microscopic examinations [16]. In addition, the molecular identification methods using the PCR to amplify a segment of the rRNA operon encompassing the 5.8S rRNA gene and the flanking internal transcribed spacers (ITS) is now in progress at the Iranian Biological Resource Center (IBRC).

### 4. Evaluation of LDPE degradation in soil

#### 4-1. Ultraviolet irradiation of polyethylene

The LDPE films were irradiated for 25 days under UV light (two 55 W lamps (Osram) made in Germany) in a laminar flow cabinet and were cut into pieces measuring approximately 3×3 cm and 15×1.5 cm for use in the biodegradation assays. For the mechanical properties analysis, the LDPE films were cut into strips with dimensions of 15×1.5 cm.

#### 4-2. Soil preparation and inoculation

The biodegradation assay was performed according to ASTM D5988**–**03 [7], a respirometric test based on the measurement of CO_2_ evolution. The soil was collected from farmlands and contained very low amounts of carbon compounds (organic carbon). The texture of the soil was loam and was composed of 35.8% sand, 26.6% clay and 37.6% silt. The organic content of the soil was 1.58% and the pH was 7.5. This pH was found to be near optimal for hydrocarbon biodegradation and it was assumed that this pH would also favour the biodegradation of plastic materials [17]. The water**-**holding capacity of the soil was determined and used to adjust the water content of the soil to 50% of the holding capacity [17], [7], [18]. The soil was sieved (<2 mm) and stored at 4°C sealed in a plastic container.

To prepare the inoculums, the fungal isolate was cultured on MEA (malt extract agar) plates and was incubated at 30°C until complete growth was obtained for the next stage. Next, 5 plugs (1×1 cm) of the fungus from the MEA plates were transferred into 50 ml Erlenmeyer flasks containing 15 ml of culture medium containing the following: (grams per litre): glucose, 10; malt extract, 10; peptone, 2; yeast extract, 2; asparagine, 1; K_2_HPO_4_, 2; MgSO_4_.7H_2_O, 1; and Thiamine**-**HCL, 0.001. The flasks were incubated at 30°C until sufficient biomass was obtained. The soil (100 g) was placed in the bottom of 2**-**litre desiccator jars. The LDPE pieces (0.1 g) were mixed with the soil and the desiccators were inoculated with the mixture of the fungus (5 ml of the fungus inoculums) and the bacterium (5 ml of a mid**-**exponential**-**phase culture of the bacterium grown in NB (nutrient broth) medium containing 1.5×10^8^ colony**-**forming units). The desiccators were incubated in a sterilised chamber at 30°C for 126 days.

The reduced water content of the soil was supplemented once per week with a mineral solution (pH 6.5) containing (grams per litre) KH_2_PO_4_, 0.7; K_2_HPO_4_, 0.7; MgSO_4_.7H_2_O, 0.7; NH_4_NO_3_, 1.0; NaCl, 0.005; MnSO_4_.7H_2_O, 0.001; ZnSO_4_.7H_2_O, 0.002; and FeSO_4_, 0.002 [6].

In this study, two main treatments were performed: soil + selected microorganisms + UV**-**irradiated LDPE films (SMUP) and soil + selected microorganisms + non**-**UV**-**irradiated LDPE films (SMP). In each treatment, three blanks were included: soil (S), soil with the selected microorganisms (SM) and soil with UV**-** or non**-**UV**-**irradiated LDPE films (SUP and SP, respectively). Each treatment was performed in triplicate.

After 126 days, the process was terminated, and the LDPE pieces were washed in distilled water, were dried and were analysed for biodegradation.

#### 4-3. Soil analyses

***Microbial count:*** The microbial population in the treatments was measured periodically (every 6 weeks) using the dilution plate method [19]. The bacteria and fungi were distinguished using different agar media containing (grams per litre) the following: for fungi, malt extract, 20; glucose, 20; peptone, 1; and for bacteria, K_2_HPO_4_, 1.0; MgSO_4_.7H_2_O, 0.02, CaCl_2_, 0.1; NaCl, 0.1; FeCl_3_, trace, KNO_3_, 0.5; asparagine, 0.5; mannitol, 1; and yeast extract, 0.25 [6]. Sterile physiological serum was used to dilute the soil samples by 10^−1^ to 10^−8^. For each of the 10^−3^, 10^−4^, 10^−5^ and 10^−6^ dilutions, 3 plates were inoculated. The plates were incubated at 28°C. The colonies were counted after an appropriate incubation time.

***Microbial biomass carbon:*** The microbial biomass carbon was measured every two weeks using the chloroform fumigation/direct extraction method for all treatments [20].

***Carbon dioxide evolution:*** To trap the evolved CO_2_, each desiccator was equipped with a beaker containing 20 ml of a 0.2 mol/L NaOH (Merck) solution, which was titrated with a 0.2 mol/L HCL (Merck) solution. The desiccators were maintained at 30°C and were opened at appropriate intervals to allow aeration and titration of the NaOH solution. Prior to the titration, 2 ml of a 0.5 mol/L BaCl_2_ solution was added to the NaOH solutions. Desiccators (3 in total) containing only the absorbing solution and no soil were also included as technical controls [7].

The percentage of biodegradation of the samples (mineralisation) was calculated taking into account the theoretical amount of carbon dioxide ([CO_2_] _Theor_) of the samples; and the percentage biodegradation: ([CO_2_] _T_*100/([CO_2_] _Theor_).

***Soil pH measurement:*** The pH of the soil was determined in a 5∶1 (distilled water: soil) slurry using a glass combination electrode calibrated with standard buffers, following the guidelines given in the Test Method ASTM D 1293**–**99, a standard test method for pH of water [21], [7].

#### 4-4. LDPE analyses

***Mechanical properties:*** The tensile strength (percentage elongation) was determined using a tensile tester (Gotech, model U 60) at room temperature and 50 mm/min with a 5**-**cm gap. The samples were equilibrated to 50% relative humidity for at least 40 h before the analysis [22].

***Fourier transform infra-red (FT-IR) analysis:*** The structural change in the LDPE surface was investigated using the EQUINOX 55 FT**-**IR spectrometer. For each LDPE film, a spectrum was taken from 400 to 4000 wavenumbers.cm^−1^. The carbonyl and double bond indices were calculated based on the relative intensities of the carbonyl band at 1,715 cm^−1^ and the double bond band at 1,650 cm^−1^ to that of the methylene scissoring band at 1,460 cm^−1^ [23].

***X-ray diffraction (XRD) analysis:*** The X-ray diffraction patterns of the films were measured with a X-ray diffractometer (D5000, Siemens Diffractometer) which is operated fully automatically using Cu Kα radiation (λ = 1.5418 A°). The scattered radiation was registered in the angular interval (2⊖) from 2°to 40°. A current of 30 mA and a voltage of 40 kV were used. All diffraction patterns were examined at room temperature and under constant operating conditions.

***Scanning electron microscopy (SEM):*** The polyethylene samples were removed from the soil and were dried in a desiccator for 24 h under vacuum. The samples were vapour**-**fixed at room temperature for three days in a sealable glass container containing two beakers, one containing 10 ml of 25% glutaraldehyde in H_2_O and the other containing 5 ml of 5% OsO_4_ in 0.1 M phosphate buffer at pH 7.0. After fixation, the container was aerated for 20 h [13]. The samples were gold**-**coated using BAL**-**TEC**-**SCDOOS and were examined using a Philips**-**X LP30 scanning electron microscope.

## Results and Discussion

### 1. Isolation and identification of the isolates

In the initial step of the isolation, 144 isolates were selected by comparing their ability to growth in solid mineral medium containing linear paraffin. In addition, the screening of these isolates was performed by comparing their growth ability in solid media containing synthetic mineral medium supplemented with 2% ethylene oligomer, resulting in the selection of 53 isolates. In total, five gram**-**positive and spore**-**forming *Bacilli* and five fungal isolates were screened based on their growth ability in a liquid mineral medium containing 5% ethylene oligomer. Of these isolates, one bacterial and one fungal isolate were selected as the final strains by comparing the growth ability in mineral medium containing PE powder as the sole source of carbon. The morphological characterisation of the fungus, including the colour of the colonies cultured on agar and the dimensions of the conidiophores and conidia, indicated that this isolate resembled *Aspergillus niger.* This strain was designated strain F1 in this study.

The taxonomic identification of the bacterial isolate (S7**–**10F), in accordance with Bergey's Manual of Systematic Bacteriology [24] and 16S rDNA sequencing indicated a 99.4% resemblance to *Lysinibacillus xylanilyticus*.

### 2. Soil microbial count

**Figure 1:**
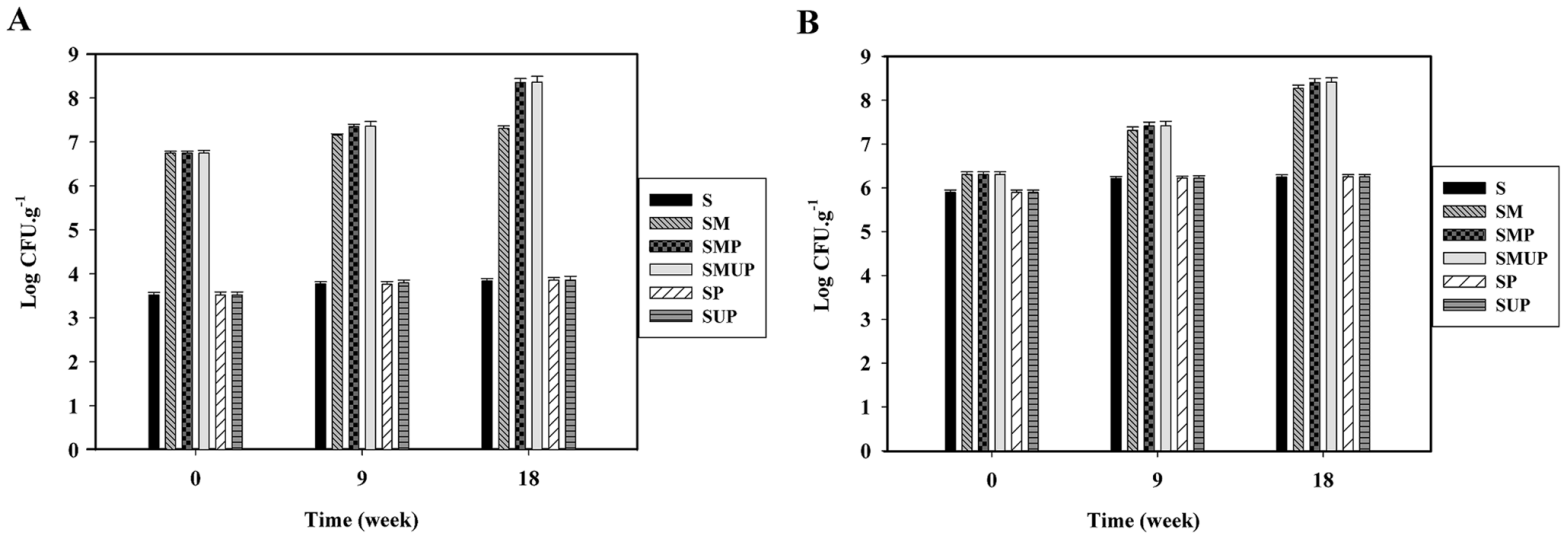
CFU count for fungal and bacterial isolates in various treatments containing LDPE films incubated in the soil for 126 day. (A) CFU count for fungal isolates in various treatments containing UV- and non-UV-irradiated pure LDPE films without pro-oxidant additives incubated in the soil for 126 days. (B) CFU count for bacterial isolates in various treatments containing UV- and non-UV-irradiated pure LDPE films without pro-oxidant additives incubated in the soil for 126 days. Each data point represents the average of three replicates ± SD. (S: Soil; SM: Soil + Selected Microorganisms; SMP: Soil + Selected Microorganisms + non-UV-irradiated PE; SMUP: Soil + Selected Microorganisms + UV-irradiated PE; SP: Soil + non-UV-irradiated PE; SUP: Soil + UV-irradiated PE).

The fungal and bacterial population was measured separately in the beginning, middle and end of the incubation period (Fig. 1A, B). As shown in Fig. 1, the microbial population increased from the beginning to the end of the incubation period for both the fungi and the bacteria, and this increment was much higher in the treatments inoculated with the selected microorganisms (SMUP, SMP and SM). These data demonstrate that soil-indigenous microorganisms cannot utilise PE as the sole carbon source (S, SP and SUP treatments). The initial fungal population of the soil was lower than the bacterial population; therefore, the difference between the fungal population in the treatments with and without the selected microorganisms, at time zero and during the incubation, was because of the growth of the selected fungal isolate, strain F1 (Fig. 1A). The bacterial population demonstrated a similar result. The difference between the un**-**inoculated treatments and the treatments inoculated with the selected bacterial isolate during the process indicated the ability of strain S7**–**10F to utilise both types of PE (UV**-**irradiated and non**-**UV**-**irradiated LDPE) as the source of carbon (Fig. 1B). For the SMUP and SMP treatments, the bacterial and fungal populations exhibited the highest number of live colonies in the 126 days, indicating the utilisation of UV**-**irradiated and non**-**UV**-**irradiated LDPE by the selected microorganisms in these treatments, respectively.

### 3. Soil microbial biomass carbon

**Figure 2:**
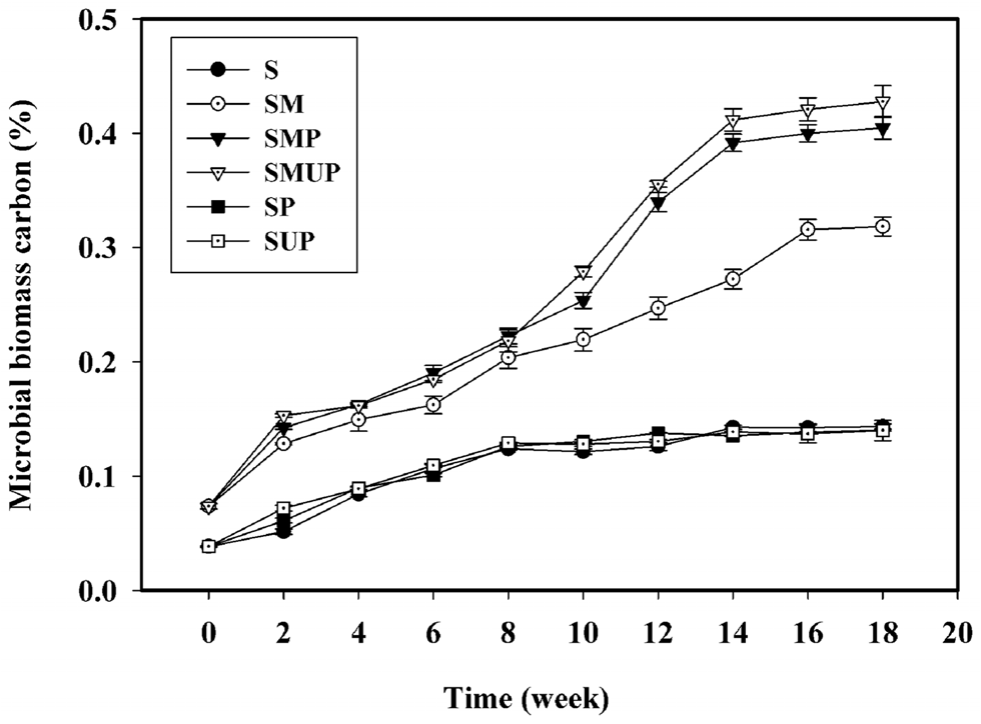
Soil microbial biomass carbon for different treatments containing UV- and non-UV-irradiated pure LDPE films incubated in the soil for 126 days. Each data point represents the average of three replicates ± SD. (S: Soil; SM: Soil + Selected Microorganisms; SMP: Soil + Selected Microorganisms + non-UV-irradiated PE; SMUP: Soil + Selected Microorganisms + UV-irradiated PE; SP: Soil + non-UV-irradiated PE; SUP: Soil + UV-irradiated PE).

The soil microbial biomass carbon (MBC) was measured every two weeks. The MBC results exhibited a similar pattern to the CFU data. The MBC increased more rapidly in the inoculated treatments (SM, SMP and SMUP, especially in the treatments containing PE as the source of carbon) than in the un**-**inoculated treatments (SUP and SP) (Fig. 2).

### 4. CO_2_ evolution

**Figure 3:**
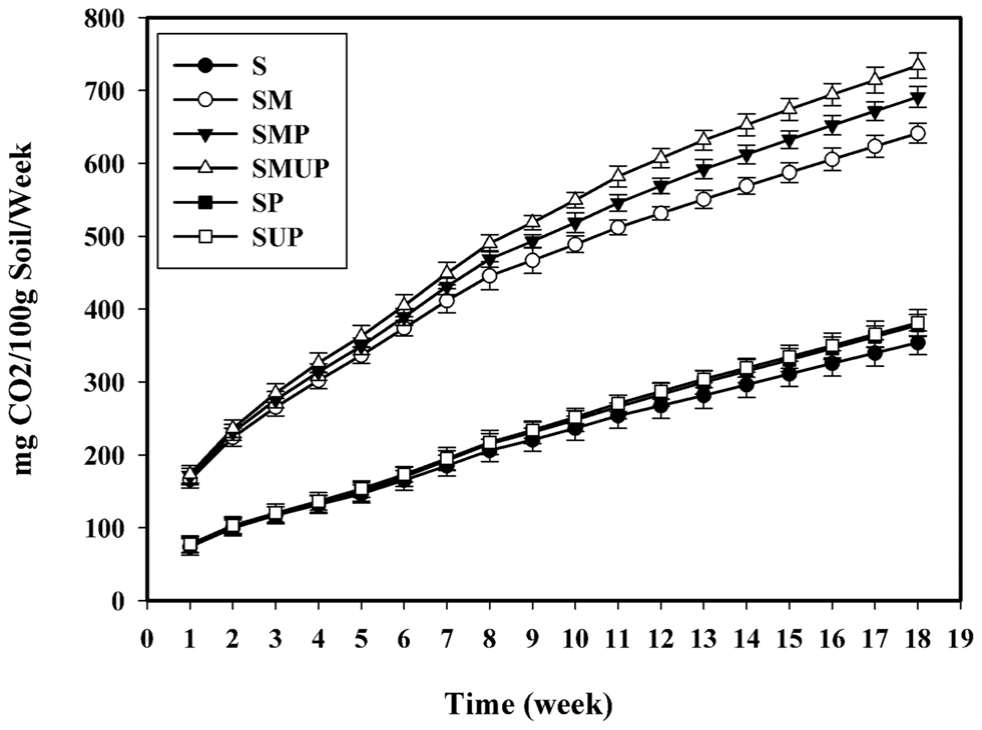
The cumulative CO2 evolution of UV- and non-UV-irradiated pure LDPE films incubated in the soil with various treatments for 126 days. Each data point represents the average of three replicates ± SD. (S: Soil; SM: Soil + Selected Microorganisms; SMP: Soil + Selected Microorganisms + non-UV-irradiated PE; SMUP: Soil+ Selected Microorganisms + UV-irradiated PE; SP: Soil + non-UV-irradiated PE; SUP: Soil + UV-irradiated PE).

**Figure 4:**
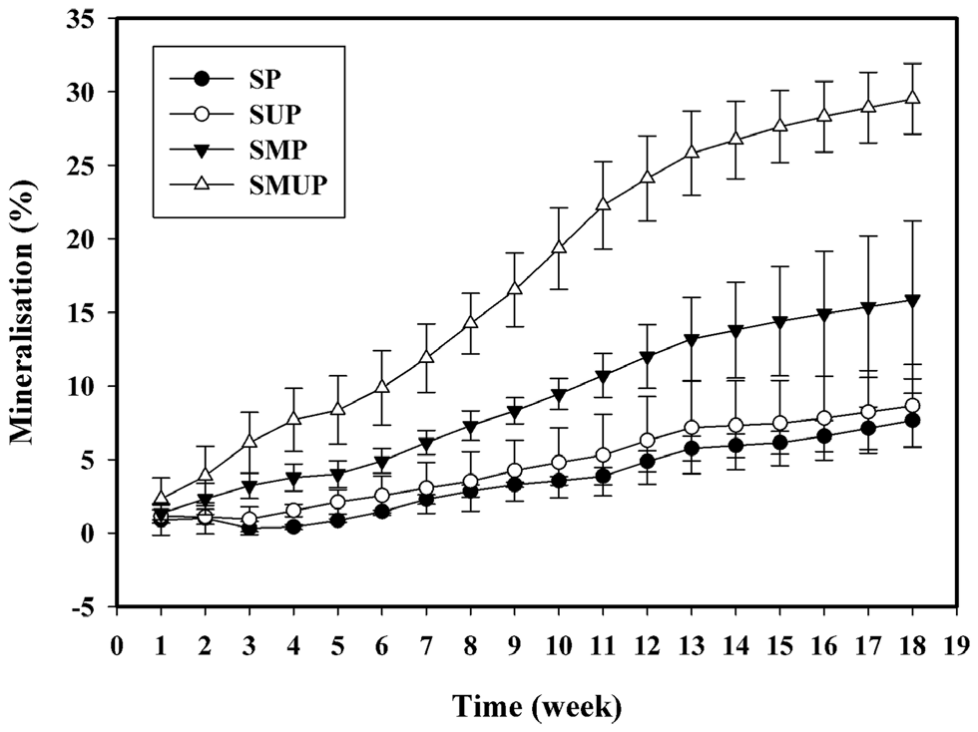
Mineralisation profile of UV- and non-UV-irradiated pure LDPE films incubated in the soil with various treatments for 126 days. Each data point represents the average of three replicates ± SD. (SP: Soil + non-UV-irradiated PE; SUP: Soil + UV-irradiated PE; SMP: Soil+ Selected Microorganisms + non-UV-irradiated PE; SMUP: Soil+ Selected Microorganisms + UV-irradiated PE).

Currently, the laboratory tests used to determine the biodegradation of plastics are based on the measurement of carbon dioxide evolution or oxygen consumption when the original polymer is exposed to controlled environmental conditions (e.g., soil, compost, active sludge, etc.). Generally, biodegradation is measured as the degree of mineralisation, namely the conversion into CO_2_. This method is considered the optimum approach for confirming the total biodegradability, i.e., the total conversion of organic carbon into inorganic carbon [25]. The amount of CO_2_ evolved was determined by the titration of the remaining NaOH. The results are presented in Fig. 3. There were no significant differences in CO_2_ evolution between the S, SP and SUP treatments. These treatments showed a slight and gradual increase in CO_2_ generation during biological degradation compared to the inoculated treatments (SMUP, SMP and SM). Of the treatments that were inoculated with the selected microorganisms, the SMUP treatment demonstrated the highest amount of CO_2_ production. The CO_2_ levels reached 734 mg CO_2_.g soil^−1^ after 126 days, which was due to the utilisation of carbonyl groups of the UV-irradiated films by the S7**–**10F and F1 strains. In addition, significant differences were observed between the SMP and SM treatments, indicating the ability of the selected microorganisms to utilise the non**-**UV**-**irradiated LDPE as the carbon source. The mineralisation profiles of the samples are shown in Fig. 4. The percentage biodegradation was higher in the inoculated treatments (SMP and SMUP) than in the un**-**inoculated treatments (SP and SUP). The mineralisation of the UV**-**irradiated LDPE films in the inoculated treatment (SMUP, 29.5%), when compared with the corresponding un**-**inoculated treatment (SUP, 8.6%), was remarkably elevated. This difference clearly demonstrates that the selected bacterial and fungal isolates utilised the oxidation products in the pre**-**oxidised films. Similar results were obtained with similar treatments containing non**-**UV**-**irradiated films (SMP and SP). The mineralisation values for pure PE without oxidation pre**-**treatment in the SMP and SP treatments were 15.8% and 7. 6% respectively. The pronounced difference between these two treatments indicates that the selected microorganisms are not only able to utilise pre**-**oxidised PE as a carbon source, as described, but can also utilise pure LDPE without oxidation pre**-**treatment.

The negligible difference in the CO_2_ production and mineralisation values between the SP and SUP treatments, and the very high level of CO_2_ generation in the SMUP and SMP treatments, clearly demonstrates the important role of the selected fungal and bacterial isolates in the biodegradation of UV**-**irradiated and non**-**UV**-**irradiated LDPE compared with the indigenous soil microbial population. Most related studies have investigated the biodegradation of abiotically aged LDPE containing pro**-**oxidant additives or PE modified with starch. The percentage mineralisation for the pre**-**oxidised LDPE and LDPE containing pro**-**oxidant additives was 16% and 24% after 317 days incubation in compost, and 9% and 12% after 317 days incubation in soil, respectively [26].

The mineralisation of thermally oxidised biodegradable LDPE after two years of incubation in soil was reported as approximately 91% [27].

Our results demonstrated higher mineralisation rates for pure LDPE without any pro**-**oxidant additives, with and without oxidation pretreatment, compared to the mineralisation rates reported by Ojeda et al. [28] for traditional LDPE without pro**-**oxidant additives exposed to sun light for 7 and 30 days. The study also reported approximately 1% mineralisation for these films after 90 days incubation in compost. Abrusci et al. [15] reported a pronounced difference between pure PE (2**–**2.5% biodegradation after 90 days) and the corresponding material containing pro**-**oxidant additives (7**–**10% biodegradation after 90 days) incubated with a mixture of *Bacillus* species.

### 5. Soil pH measurement

**Figure 5:**
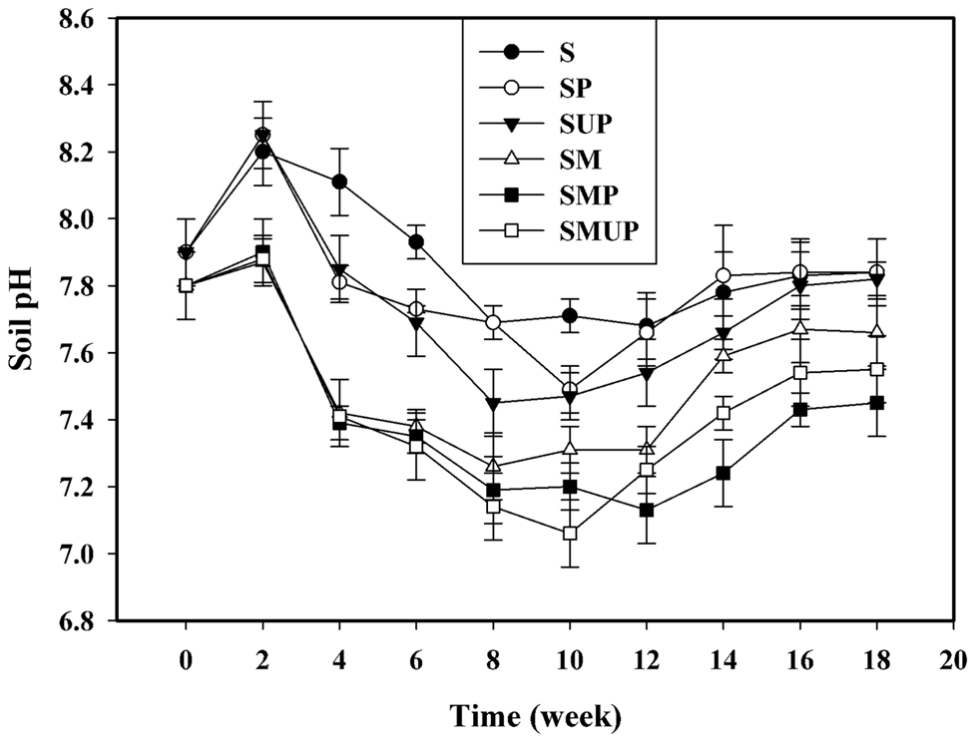
pH changes in inoculated, un-inoculated and blank soil samples for UV- and non-UV-irradiated pure LDPE films. Each data point represents the average of three replicates ± SD. (S: Soil; SM: Soil + Selected Microorganisms; SMP: Soil + Selected Microorganisms + non-UV-irradiated PE; SMUP: Soil+ Selected Microorganisms + UV-irradiated PE; SP: Soil + non-UV-irradiated PE; SUP: Soil + UV-irradiated PE).

The pH value is a key factor for the survival and activity of microorganisms. Generally, the pH should be between 6 and 8 [7]. The periodic measurements of the soil pH are presented in Fig. 5. The initial increase in the pH values may be because of the ammonification of nitrogen components [11]. The decrease and increase in pH was also reported in a study by Jakubowicz et al. [27] in which the biodegradation of thermally oxidised biodegradable LDPE in soil for a period of 606 days was evaluated.

### 6. Mechanical properties of polyethylene films

The mechanical properties of the non**-**UV**-** and UV**-**irradiated films are shown respectively in Tables 1 and 2, during and after the biodegradation process. There was no significant difference (P = 0.05) in percentage elongation between time zero and after 63 days' incubation of non-UV-irradiation films in the absence of the selected microorganisms (Table 1, treatment SP). The elongation at break (%) of the non**-**UV**-**irradiated LDPE films decreased 8.9% in the un**-**inoculated treatment (SP) after 126 days' incubation, whereas the percentage elongation of these films decreased 17.4% and 48% in the inoculated treatment (SMP) after 63 and 126 days' incubation, respectively (Table 1). Reductions in elongation of 17.8% and 83% were recorded after 63 days for the UV**-**irradiated LDPE films in the un**-**inoculated (SUP) and inoculated (SMUP) treatments, respectively (Table 2).

**Table 1:** Changes in the percentage elongation of non-UV-irradiated PE films before and after 63 and 126 days of biodegradation in soil in various treatments.

Treatments	Time 0	Week 9		Week 18	
	ε_r_ ± SD	ε_r_ ± SD	(Δ%)	ε_r_ ± SD	(Δ%)
**SP**	299.5±6.2 a	297.6±9.7 a	0.6 (−)	272.9±7 b	8.9 (−)
**SMP**	299.5±6.2	247.3±13.3 c	17.4 (−)	155.5±11.8 d	48 (−)

*ε_r_* elongation at break (%), (Δ %) difference between percentage elongation of films before and after biodegradation process (shown as a percentage). Values accompanied by a similar letter are not significantly different according to Duncan's multiple- range test (P = 0.05). Each value represents the average of four replicates ± SD. (SP: Soil + non-UV-irradiated PE; SMP: Soil + Selected Microorganisms + non-UV-irradiated PE).

**Table 2:** Changes in the percentage elongation of UV-irradiated PE films before and after 63 and 126 days of biodegradation in soil in various treatments.

Treatments	Time 0	Week 9		Week 18	
	ε_r_ ± SD	ε_r_ ± SD	(Δ%)	ε_r_ ± SD	(Δ%)
**SUP**	12.6±1.2 a	10.3±1.2 b	17.8 (−)	*	−
**SMUP**	12. 6±1.2	2.1±0.4 c	83 (−)	*	−

*ε_r_* elongation at break (%), * fragile specimens, (Δ %) difference between percentage elongation of films before and after biodegradation process (shown as a percentage). Values accompanied by a similar letter are not significantly different according to Duncan's multiple range test (P = 0.05). Each value represents the average of four replicates ± SD. (SUP: Soil + UV-irradiated PE; SMUP: Soil+ Selected Microorganisms + UV-irradiated PE).

The long polymer chains were likely cut into shorter pieces because of the action of enzymes secreted by the microorganisms. Because the films became fragile and lighter in weight indicates the preliminary stages of microbial decomposition, consisting of a reduction in molecular weight [29].

The UV irradiation of the films caused a reduction in the percentage elongation of 95. 8%. This result is in agreement with a study by Lee et al. [30] reporting an increase in the percentage elongation of degradable films after 2 weeks of UV irradiation, and a marked decrease in this parameter after 4 and 8 weeks of treatment. The percentage elongation of 8**-**week UV**-**irradiated degradable films was near to zero. Two-week UV**-**irradiated degradable films demonstrated a reduction in the percentage elongation after 4 weeks' of incubation in culture media containing ligninolytic microorganisms. A reduction in the percentage elongation of the LDPE film after thermal oxidation was reported by Jakubowicz et al. [27]. In addition, Orhan and Buyukungor [6], Jakubowicz et al. [31] and Nowak et al. [29] reported a reduction in the percentage elongation of polyethylene films after the biodegradation process.

### 7. FT-IR analysis

**Figure 6:**
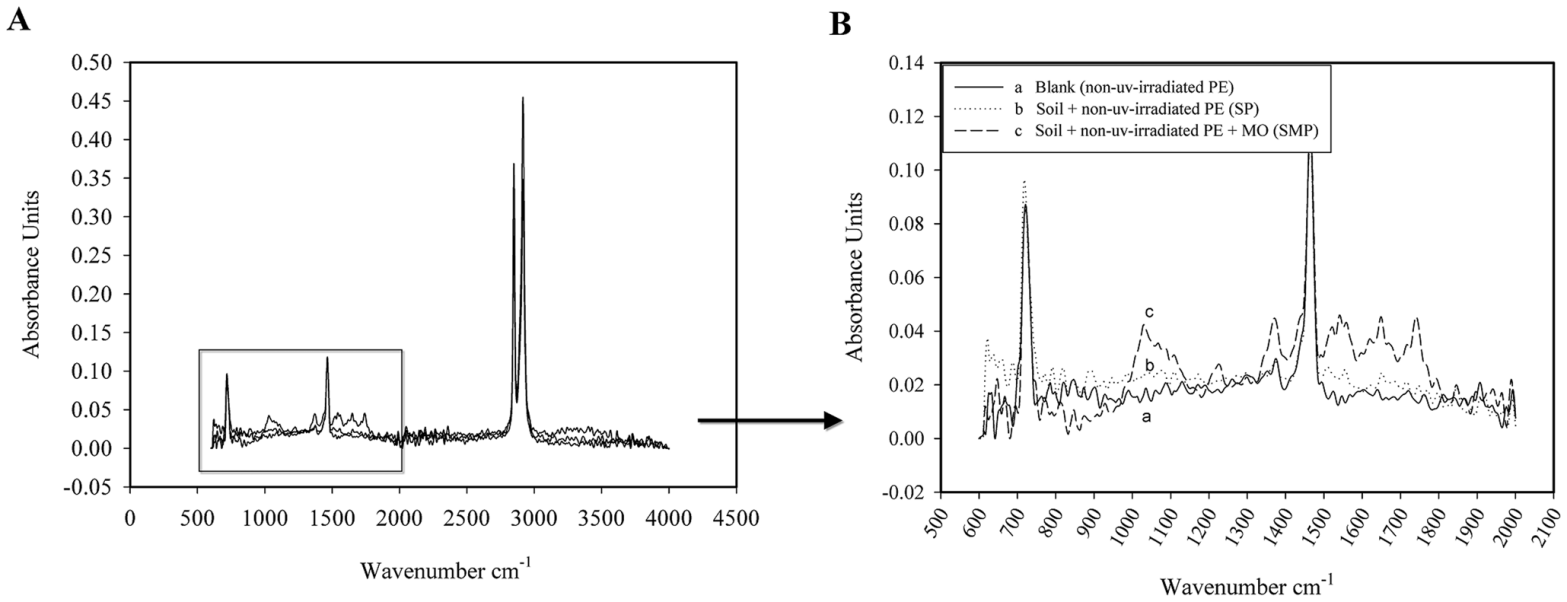
FT-IR spectra of non-UV-irradiated pure LDPE films before and after incubation in soil in various treatments. (A) FT-IR spectra of non-UV-irradiated pure LDPE films without pro-oxidant additives before and after 126 days of incubation in soil in the presence and absence of the selected microorganisms from 500–4000 cm-1. (B) The changes in the bands between 500 and 2,000 cm-1 of the FT-IR spectra of non-UV-irradiated pure LDPE films without pro-oxidant additives before and after 126 days of incubation in soil with different treatments: (a) blank (no UV irradiation, no incubation); (b) non-UV-irradiated LDPE after incubation in soil in the absence of the selected microorganisms (SP treatment); (c) non-UV-irradiated LDPE after incubation in soil in the presence of the selected microorganisms (SMP treatment).

**Figure 7:**
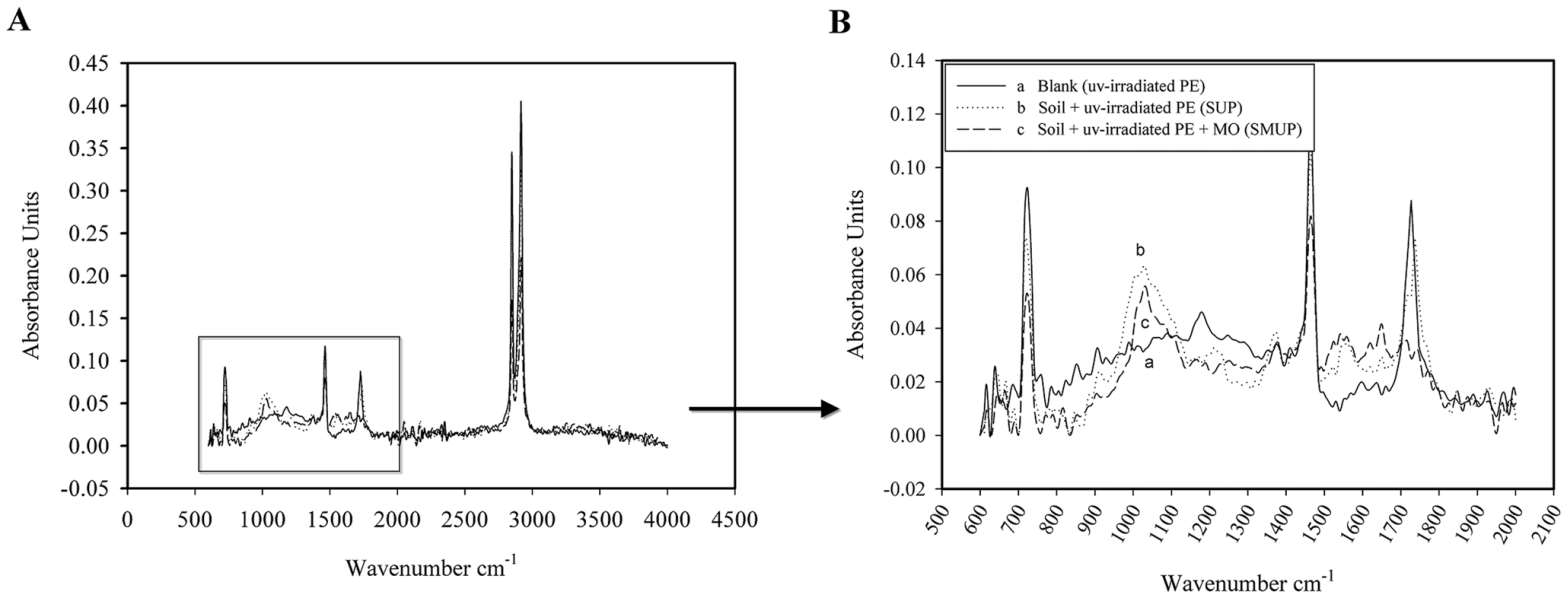
FT-IR spectra of UV-irradiated pure LDPE films before and after incubation in soil in various treatments. (A) FT-IR spectra of UV-irradiated pure LDPE films without pro-oxidant additives before and after 126 days of incubation in soil in the presence and absence of the selected microorganisms from 500–4000 cm-1. (B) The changes in the bands between 500 and 2,000 cm-1 of the FT-IR spectra of UV-irradiated pure LDPE films without pro-oxidant additives before and after 126 days of incubation in soil with different treatments: (a) blank (after 25 days' UV irradiation, no incubation); (b) UV-irradiated LDPE after incubation in soil in the absence of the selected microorganisms (SUP treatment); (c) UV-irradiated LDPE after incubation in soil in the presence of the selected microorganisms (SMUP treatment).

In the biodegradation of polyethylene, the initial abiotic step involves the oxidation of the polymer chain leading to the formation of carbonyl groups. These groups eventually form carboxylic groups, which subsequently undergo β**-**oxidation [23] and are completely degraded via the citric acid cycle resulting in the formation of CO_2_ and H_2_O. β**-**oxidation and the citric acid cycle are catalysed by microorganisms. Monitoring the formation and disappearance of carbonyl and double bond bands using FT-IR is necessary to elucidate the mechanism of the biodegradation process. Figure 6 shows the FT-IR spectra of non**-**UV**-**irradiated pure LDPE films without pro**-**oxidant additives before and after the 126 days of incubation in soil in the presence and absence of the selected microorganisms. FT-IR spectra of non**-**UV-irradiated LDPE films before and after 126 days of incubation in soil in the presence and absence of the selected microorganisms from 500–4000 cm^−1^ is shown in Fig. 6A. The changes in the bands between 500 and 2,000 cm^−1^ are magnified in Fig. 6B. The FT-IR spectra of the LDPE film without oxidation pretreatment is shown in Fig. 6B.a. The spectrum of the non**-**UV**-**irradiated LDPE film, which is introduced into soil in the absence of the selected microorganisms (SP treatment), is shown in Fig. Fig. 6B.b. Compared with the corresponding control, significant changes in the spectra of the non**-**UV**-**irradiated LDPE film after 126 days incubation were not observed (Fig. 6B.a, no UV irradiation, no incubation). The spectrum of this film, incubated in soil that was inoculated with the selected microorganisms, shows the appearance of several new bands (Fig. 6B.c, SMP treatment). The carbonyl absorption bands can be observed in the range of 1,710−1,750 cm^−1^ because of the formation of ketone or aldehyde C = O groups by the action of the selected microorganisms. The absorbance in the region of 1,541 cm^−1^ is associated with the carboxylate group [32]. Additionally, new absorption bands between 1,000 and 1,700 cm^−1^ (1,029 and 1,371 cm^−1^) of the spectra are possibly due to the oxidised fractions, such as moieties containing –OH groups, resulting from biodegradation by the selected microorganisms [33]. The selected bacterial and fungal isolates were capable of utilising long, hydrophobic polyethylene chains. In our study, the selected microorganisms from the landfill source oxidised even virgin LDPE without oxidation pretreatment and pro-oxidant additives. These results are in contrast to many reports in which the microorganisms could assimilate only the products of pre**-**oxidised PE [34], [11]. Because the initial breakage of PE chains is the longest and most difficult step in its degradation, long incubation times produce significant quantities of carbonyl groups to continue the decomposition process [29]. Figure 7 shows the FT-IR spectra of the UV**-**irradiated pure LDPE films without pro**-**oxidant additives before and after the 126 days of incubation in soil in the presence and absence of the selected microorganisms. FT-IR spectra of the UV-irradiated LDPE films before and after 126 days of incubation in soil in the presence and absence of the selected microorganisms from 500–4000 cm^−1^ is shown in Fig. 7A. The changes in the bands between 500 and 2,000 cm^−1^ are magnified in Fig. 7B. The FT-IR spectra of the LDPE film after 25 days of UV irradiation (Fig. 7B.a) shows the appearance of a peak in the range of 1,710**–**1,750 cm^−1^ due to the formation of carbonyl groups (abiotic oxidation). The intensity of the bands in the 1,178 cm^−1^ region is increased and is related to carbonyl groups [10]. Figure 7B.b shows the FT-IR spectrum of the UV**-**irradiated LDPE film incubated in un**-**inoculated soil (SUP treatment). The new absorption bands between 1,000- and 1,700 cm^−1^ (1028 and 1373 cm^−1^) are also because of the oxidised fractions, such as moieties containing –OH groups resulting from the action of the indigenous soil microorganisms [33]. The comparison of the spectra from the SP and SUP treatments (Figs. 6B.b and 7B.b) shows clearly that the indigenous soil microorganisms could not utilise the virgin PE with a hydrophobic nature but were capable of attaching to and partially oxidising the UV**-**irradiated LDPE film.

The spectrum of the UV**-**irradiated LDPE film which is introduced into soil in the presence of the selected microorganisms (SMUP treatment), is shown in Fig. 7B.c. Compared with the corresponding control, the intensity of the carbonyl band at 1,710**–**1,750 cm^−1^ was significantly decreased during the process with the selected microorganisms. The intensity of the bands in the 1,000**–**1,700 cm^−1^ range (1,071, 1,541 and 1,649 cm^−1^) is also attributed to the oxidised fractions because of the action of the selected microorganisms. Part of the decreased absorption at 1,714 cm^−1^ is compensated by the appearance of carboxylates at 1,541 cm^−1^ (Fig. 7B.c, SMUP treatment) [32]. The indigenous soil microorganisms reduced the carbonyl index (CI) 5.6% (Table 3 and SUP treatment in Fig. 7B.b), whereas selected microorganisms reduced the CI 42% (Table 3 and SMUP treatment in Fig. 7B.c).

**Table 3:** Carbonyl and double bond indices values determined using FTIR from LDPE films before and after 126 days incubation in soil in various treatments.

Treatments	Time 0		Week 18		(Δ%)	
	CI	DBI	CI	DBI	CI	DBI
**SP**	0.151	0.154	0.187	0.216	23.8 (+)	40.2 (+)
**SUP**	0.747	0.168	0.705	0.280	5.6 (−)	66.7 (+)
**SMP**	0.151	0.154	0.401	0.401	165. 6 (+)	160.4 (+)
**SMUP**	0.747	0.168	0.433	0.507	42 (−)	201. 8 (+)

*CI* carbonyl index, *DBI* double bond index, (Δ%) difference between carbonyl and double bond indices of films before and after 126 days of biodegradation (shown as a percentage). (SP: Soil + non-UV-irradiated PE; SUP: Soil + UV-irradiated PE; SMP: Soil + Selected Microorganisms + non-UV-irradiated PE; SMUP: Soil + Selected Microorganisms + UV-irradiated PE).

An increase in the double bond index (DBI) was observed in all treatments and was especially significant in the SMP and SMUP treatments (Table 3). The decrease in the CI in the SMUP and SUP treatments and the increase in the DBI in all samples, especially the SMP and SMUP treatments, can be explained using the proposed mechanism for PE biodegradation. According to this mechanism, formed carbonyl groups along the polymeric chain, resulting from the action of abiotic factors, can be attacked microbially (CI decrease), and lead to the release of unsaturated chains (DBI increase) [9]. The oxidised group is transformed to a carboxylic acid and is metabolised via β**-**oxidation.

Manzur et al. [10] reported that the segments formed by the rupturing of the chains because of the biological treatment could cause the formation of the vinyl group and the increase in the DBI. In addition, the increase in the DBI can be attributed to biotic dehydrogenation [35].

### 8. XRD analysis

**Figure 8:**
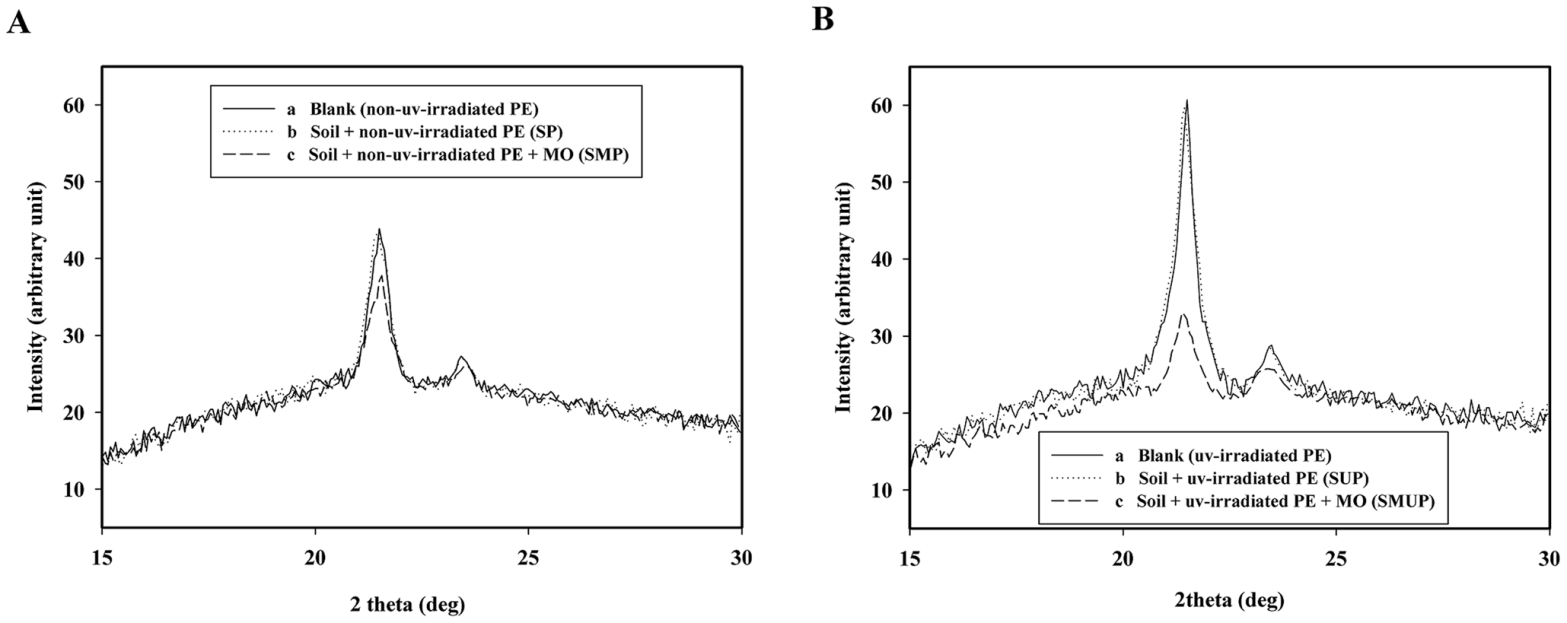
XRD spectra of non-UV and UV-irradiated pure LDPE films before and after incubation in soil with different treatments. (A) XRD spectra of non-UV-irradiated pure LDPE films without pro-oxidant additives before and after 126 days of incubation in soil with different treatments: (a) blank (no UV irradiation, no incubation); (b) non-UV-irradiated LDPE after incubation in soil in the absence of the selected microorganisms (SP treatment); (c) non-UV-irradiated LDPE after incubation in soil in the presence of the selected microorganisms (SMP treatment). (B) XRD spectra of UV-irradiated pure LDPE films without pro-oxidant additives before and after 126 days of incubation in soil with different treatments: (a) blank (after 25 days' UV irradiation, no incubation); (b) UV-irradiated LDPE after incubation in soil in the absence of the selected microorganisms (SUP treatment); (c) UV-irradiated LDPE after incubation in soil in the presence of the selected microorganisms (SMUP treatment).

The XRD spectra of the non**-**UV**-** and UV-irradiated pure LDPE films is shown in Fig. 8, before and after 126 days of incubation in soil in the presence and absence of the selected microorganisms. As shown in this figure, the XRD spectra show distinguished peaks at 21.4 and 23.5 of the angular position 2⊖. The intensity of the peaks of UV-irradiated films is higher than that of non**-**UV-irradiated one (Fig. 8A.a and Fig. 8B.a). This difference clearly demonstrated that oxidation pretreatment increased the degree of polyethylene crystalinity.

The intensity of the peaks was significantly decreased after 126 days of incubation in soil in the presence of the selected microorganisms (Fig. 8A.c, SMP treatment and Fig. 8B.c, SMUP treatment). There were no significant differences in degree of crystalinity between corresponding controls and the films incubated in soil in the absence of the selected microorganisms (Fig. 8A.b, SP treatment and 8A.a, Fig. 8B.b, SUP treatment and 8B.a). These results indicated that crystalinity and the crystal sizes for non-UV-irradiated and UV-irradiated films decreased during process with the selected microorganisms [36].

### 9. Scanning electron microscopy analysis

**Figure 9:**
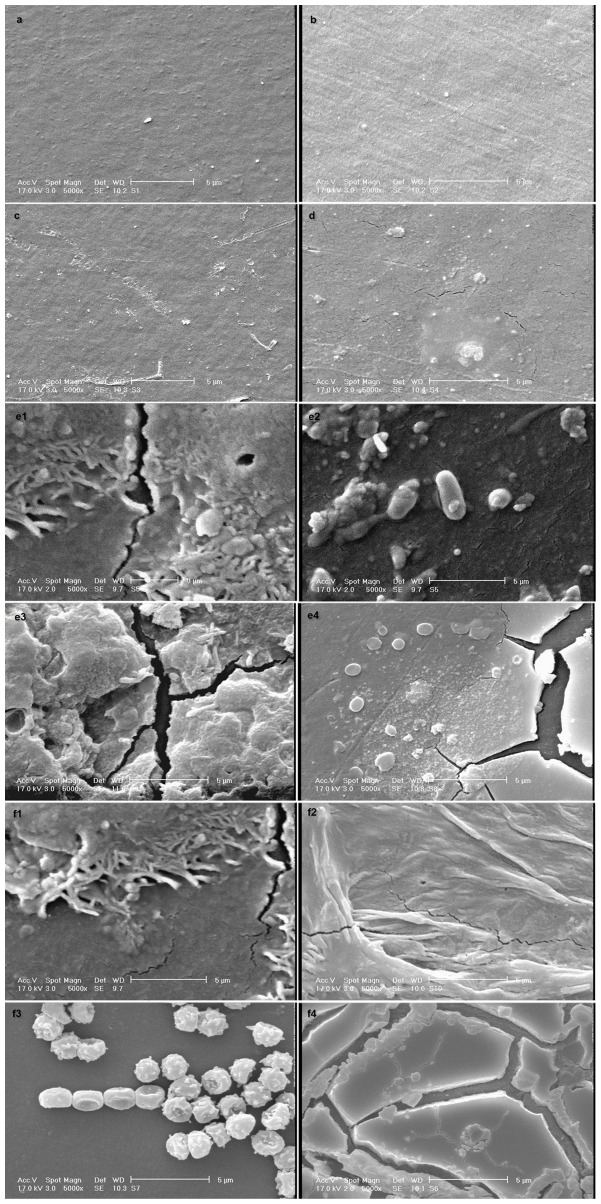
SEM micrograph of pure LDPE films before and after 126 days of incubation in soil with different treatments. (a) Blank (no UV irradiation, no incubation). (b) UV-irradiated LDPE film without incubation. (c) non-UV-irradiated LDPE film incubated in soil in the absence of the selected microorganisms (SP). (d) UV-irradiated LDPE film incubated in soil in the absence of the selected microorganisms (SUP). (e) non-UV-irradiated LDPE film incubated in soil in the presence of the selected microorganisms (SMP): (e1) penetration of hyphae into the LDPE matrix; (e2) formation of bacterial biofilm on the surface of LDPE; (e3 and e4) formation of pits and cavities on the surface of LDPE. (f) UV-irradiated LDPE film incubated in soil in the presence of the selected microorganisms (SMUP): (f1 and f2) penetration of hyphae into the LDPE matrix; (f3) formation of bacterial biofilm on the surface of LDPE; and (f4) formation of pits and cavities on the surface of LDPE.

The SEM analysis was performed to monitor the changes in the surface of the films. The adhesion of the microorganisms to the polymeric surface is fundamental for biodegradation to take place [9]. Figure 9 shows the SEM micrographs of the PE surfaces before and after 126 days of incubation with the different treatments. Before processing, the samples had a smooth surface with no defects (Figs. 9a and 9b). In addition, no special features were detected in the SEM micrograph of the films introduced into the soil without the selected microorganisms (Figs. 9c and 9d). However, after incubation with the selected microorganisms, surface erosion and the formation of pits and cavities on the surface of the samples can be observed (Figs. 9e and 9f). The presence of pits and cavities may be because of the absence of a uniform distribution of short branches or photodegradable products in the polymer matrix [10], suggesting that the fungus (strain F1) penetrated into the LDPE matrix during degradation. This penetration of hyphae into the matrix, and the formation of a bacterial biofilm of the strain S7**–**10F on the surface of the films were observed in both the (UV- and non**-**UV**-**irradiated PE) incubated in the soil with the selected microorganisms (SMP and SMUP treatments).

The LDPE degradation by *Aspergillus niger* and *Aspergillus fumigatus* is consistent with the results obtained previously [9], [10], [11]. *Aspergillus terreus* also participated in the degradation of modified and unmodified PE [12]. Moreover, there are examples in the literature confirming the ability of the genus *Bacillus* to degrade PE. *Bacillus pumilus* and *B. halodenitrificans* were able to degrade an abiotically aged LDPE containing pro**-**oxidant within 120 days [37]. *Bacillus sphericus* and *B. cereus* have also been shown to degrade LDPE and HDPE unmodified and modified with starch [34]. The biodegradation of photo**-**degraded LDPE containing pro**-**oxidant additives by a mixture of *Bacillus* strains (*B. megaterium, B. subtilis* and *B. cereus*) was evaluated within 90 days, and biofilm formation developed only in the photo**-**degraded material after one week of the bacterial treatment [15].

## Conclusion

In this study, the biodegradation of pure LDPE films without pro**-**oxidant additives, with and without photo**-**oxidation pretreatment, was evaluated in soil in the presence and absence of a mixed culture of selected landfill**-**source microorganisms (*Aspergillus niger* designated strain F1 and *Lysinibacillus xylanilyticus* XDB9 (T) designated strain S7**–**10F). The data obtained from the respiration and microbial population measurements showed significant differences between the inoculated and un**-**inoculated treatments with the selected microorganisms. The carbon dioxide measurements showed that the biodegradation in the un**-**inoculated treatments were slow and were about 7.6% and 8.6% of mineralisation for the non**-**UV**-**irradiated and UV**-**irradiated LDPE respectively after 126 days. In contrast, in the presence of the selected microorganisms, the biodegradation was much more efficient and the percentages of biodegradation were 29.5% and 15.8% for the UV**-**irradiated and non**-**UV**-**irradiated films, respectively. The percentage decrease in the CI was higher for the UV**-**irradiated LDPE when the biodegradation was performed in soil inoculated with the selected fungal and bacterial isolates. The FT-IR, XRD and SEM analyses demonstrated the ability of the selected microorganisms to modify and colonise both types of PE as the carbon source, and demonstrated the important role of these isolates in the PE biodegradation process. The oxidation pretreatment facilitated the biodegradation of PE; however, contrary to other reports, our study confirmed the ability of the selected fungal and bacterial isolates to utilise virgin PE without pro**-**oxidant and oxidation pretreatments. The results of this study show that the selected microorganisms (strains S7**–**10F and F1) exhibit great potential for LDPE biodegradation under natural conditions, such as those found in soil. In the near future, these microorganisms can be used to reduce the quantity of solid waste, which is rapidly accumulating in the natural environment.
